# Update on Diagnosis and Management of Onychophagia and Onychotillomania

**DOI:** 10.3390/ijerph19063392

**Published:** 2022-03-13

**Authors:** Debra K. Lee, Shari R. Lipner

**Affiliations:** 1 Paul L. Foster School of Medicine, Texas Tech University Health Sciences Center El Paso, El Paso, TX 79905, USA; debra.lee@ttuhsc.edu; 2 Department of Dermatology, Weill Cornell Medicine, New York, NY 10021, USA

**Keywords:** body-focused repetitive behavior, BFRB, onychophagia, nail biting, onychotillomania, nail picking

## Abstract

Onychophagia (nail biting) and onychotillomania (nail picking) are chronic nail conditions categorized as body-focused repetitive behavior (BFRB) disorders. Due to a limited awareness of their clinical presentations, embarrassment on the part of patients, and/or comorbid psychiatric conditions, these conditions are frequently underrecognized and misdiagnosed. This article reviews the prevalence, etiology, diagnostic criteria, historical and physical exam findings, and treatment options for these conditions. The PubMed/MEDLINE database was searched for relevant articles. Onychophagia and onychotillomania are complex disorders necessitating a detailed patient history and physical examination and a multidisciplinary treatment approach for successful diagnosis and management. Due to the dearth of clinical trials for treatment of nail biting and nail picking, large clinical trials are necessary to establish standardized therapies.

## 1. Introduction

Onychophagia, or habitual nail biting, and onychotillomania, or repetitive nail picking and pulling, are chronic nail conditions categorized as body-focused repetitive behavior (BFRB) disorders. Both disorders can cause damage to the nail matrix, nail bed, nail plate, and periungual skin, creating physical and psychosocial consequences. Although nail biting and nail picking are relatively common in the general population, particularly in children and individuals under stress, limited awareness of their clinical presentations, feelings of shame toward the habit, failure to refer to mental health specialists, and coexisting psychiatric conditions can contribute to delayed diagnosis and treatment. Accurate diagnosis involves careful history and physical examination, as patients rarely present with nail biting or nail picking as the chief complaint. Successful management of onychophagia and onychotillomania involves non-pharmacological and pharmacological treatments, necessitating a multi-disciplinary approach involving dermatologists, internists, pediatricians, psychiatrists, and dentists.

While there is a growing interest in BFRB disorders, onychophagia and onychotillomania still receive less attention in the psychiatric and dermatologic literature compared to other self-inflicted dermatoses, like skin picking and hair pulling. This has created gaps in knowledge for recognizing these nail conditions, including delayed diagnosis, and non-evidence-based treatments. Previous reviews of onychophagia and onychotillomania are limited in terms of descriptions of diagnosis and management or are outdated. Therefore, this review describes the clinical characteristics, psychiatric co-morbidities, and current treatments for onychophagia and onychotillomania.

## 2. Materials and Methods

**Figure 1:**
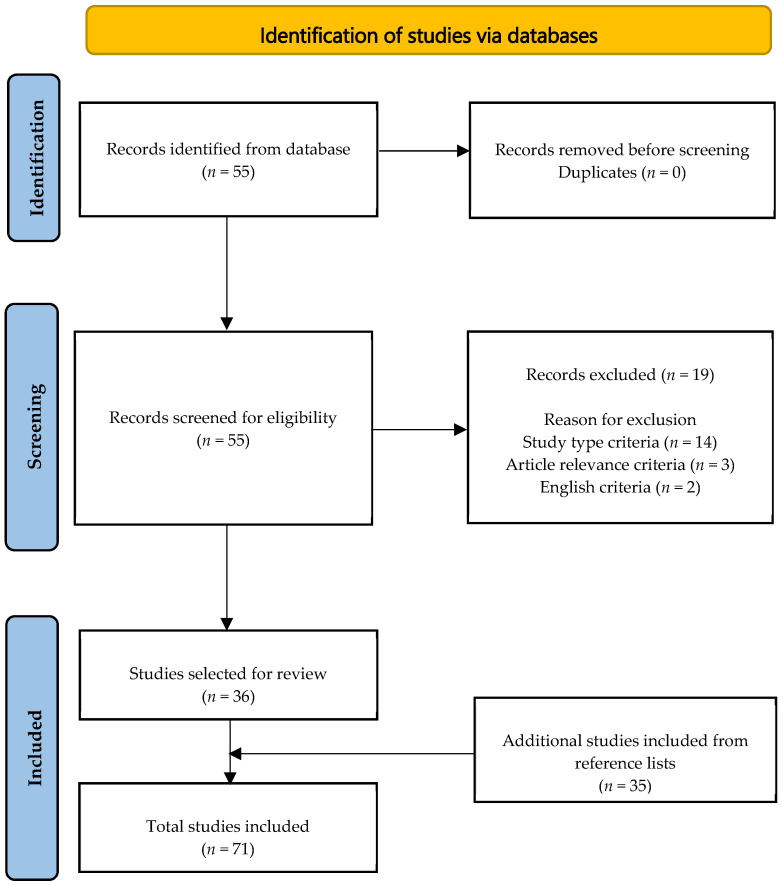
PRISMA flowchart for literature search.

Searches for peer-reviewed articles were conducted using the PubMed/MEDLINE database with the following search terms: (onychophagia OR “nail biting”[MeSH] OR onychotillomania OR “nail picking”) AND (skin diseases OR “skin disease”[MeSH] OR dermatology OR “dermatology”[MeSH]) AND (treatment OR “treatment”[MeSH]). Full length articles of randomized controlled trials, uncontrolled trials, systematic reviews, cross-sectional studies, cohort studies, case-controlled studies, case reports, and case series were included in this review. Articles that discussed clinical presentations, diagnosis, associated psychiatric disorders, and management for onychophagia and onychotillomania were included. The search was limited to articles published in the English language. Reasons for exclusion are shown in the PRISMA flowchart (Figure 1). A total of 36 articles were selected for review. Reference lists from these articles were used to find an additional 35 articles, such that a total of 71 were analyzed for this review.

## 3. Onychophagia and Onychotillomania

### 3.1. Onychophagia

#### 3.1.1. Overview and Prevalence

Onychophagia is defined as chronic biting of the nail plate, nail folds, nail bed, and/or cuticle. It is estimated to affect up to 20–30% of the general population [1,2,3] and up to 45% of children ages 10 years to puberty [4]. The rate of nail biting decreases with age, though some continue or begin the habit in adulthood [5,6]. Populations under undue stress, particularly university students, are also prone to this behavior. The prevalence is likely underestimated as patients may feel shame, and therefore avoid seeking medical evaluation. Other patients may not recognize nail biting as a medical condition that can be effectively managed or know that physicians are resources for management.

#### 3.1.2. Psychiatric Classification

Onychophagia is classified in the *Diagnostic and Statistical Manual of Mental Disorders, 5th edition* (DSM-5) under the “Other Specified Obsessive-Compulsive and Related Disorders” subcategory. The DSM-5 further characterizes onychophagia as a recurrent BFRB disorder, along with lip biting and cheek chewing. The diagnostic criteria are met when patients experience clinically significant distress or impairment in social and occupational areas of functioning, that is not better explained by trichotillomania (hair pulling), excoriation disorder (skin picking), stereotypic movement disorder, or nonsuicidal self-injury [7]. These patients must also have repeated failed attempts to suppress their nail biting [1,7].

#### 3.1.3. Psychiatric Comorbidities

Despite onychophagia falling under the umbrella of obsessive-compulsive disorder (OCD), there is currently inconsistent data comparing the association of chronic nail biting and OCD. One study found only 25% of nail biters suffered from comorbid OCD or anxiety disorder. In addition, a prevalence of 3.1% for OCD in nail biters was reported, which is similar to the lifetime prevalence in the general population [1,2].

Several studies investigating onychophagia have shown an association with other coexisting psychiatric conditions [6,8,9,10]. In a study of 63 nail biters, ages 5 to 18 years, who were referred to a child and adolescent mental health clinic, attention deficit hyperactive disorder (74.6%), oppositional defiant disorder (36%), separation anxiety disorder (20.6%), enuresis (15.6%), tic disorder (12.7%), and OCD (11.1%) were the most common conditions associated with onychophagia [6].

Another pediatric study of 147 students, ages 8 to 14 years, evaluated symptoms of anxiety and depression in children with deleterious oral habits (DOH) using the Revised Children’s Manifest Anxiety Scale and Children’s Depression Inventory. Nail biting was the most common DOH (58.7%). The habit-free group average score for depression (7.5) was significantly lower than the habit group score (10.4), suggesting that patients with DOH presented with more depressive symptoms (*p* < 0.05). There was also an association between anxiety symptoms and presence of DOH (OR = 2.35; *p* < 0.05) [11].

One study analyzed parents’ responses to questionnaires assessing mental health and nail-biting habits of 743 children. A Strengths and Difficulties Questionnaire assessed for hyperactivity/inattention, emotional symptoms, conduct problems, peer relationship problems, and prosocial behavior (a child’s ability to voluntarily act in a positive, helpful, and cooperative manner). A total of 166 (22.3%) nail biters were identified and their scores were compared to those of non-biters. Nail biters, on average, had significantly higher emotional symptom (4.3) and conduct problem scores (3.2) and lower prosocial behavior scores (7.6) compared to those of non-biters (3.7, 2.9, and 8.15, respectively) [8].

In a sample of 3475 undergraduate and high school students, onychophagia was more common in individuals with personal (32.5%) or family histories (29.7%) of psychiatric disorders compared to those without (16.4%, 16.3%, respectively) [10].

#### 3.1.4. Etiology

The etiology of onychophagia is unknown and likely multifactorial. There is thought to be a strong genetic component. One survey-based study of 281 participants at an outpatient pediatric clinic, ages 3 to 21, reported that a majority (63%) of nail-biting patients had at least one family member with onychophagia [9]. Similarly, the previously mentioned survey-based study analyzing mental health and nail-biting habits in 743 children reported that 55.8% of nail biters with one or more siblings had at least one sibling or parent who bit their nails frequently [8].

In a study analyzing maternal responses to a questionnaire about their twins (*n* = 1131 twin pairs), nail biting was attributable to genetic influences for 50% of twin pairs, with concordance rate higher for monozygotic compared to dizygotic pairs. In addition, nail biters with a history of nail biting in both their parents had a 3- to 4-fold higher risk of nail biting compared to children whose parents did not bite their nails [12].

In addition to genetic influences, environmental factors such as imitating parental or sibling behavior may contribute to the development of onychophagia [10]. Therefore, it may be beneficial to counsel patients and evaluate family members of nail biters for similar habits.

#### 3.1.5. Contributing Factors and Quality of Life

Onychophagia may be due to stress, boredom, or inactivity. For some individuals, onychophagia is an automatic behavior, meaning they unconsciously bite their nails during activities like waiting in line or reading a book. While for others, nail biting is intentional and they will often cease other activities to bite their nails [13]. Onychophagia sufferers may report a build-up of tension trying to resist urges to bite, followed by relief or pleasure after biting. These patients are more likely to also have general anxiety disorder [2]. One observational study explored the frequencies of nail biting in 40 undergraduate nail biters under 4 settings (alone; noncontingent social interaction, in which participants had conversation on neutral topics; academic demand, in which participants completed 20 math questions; and social disapproval, in which participants were reprimanded for nail biting). On average, students bit their nails more often when they were alone (6.48 times) and during academic demand (3.15 times) (chi-square = 84.1, df = 3, *p* < 0.001). Nail biting improved with noncontingent social interaction (0.25 times) and with social disapproval (0.20 times) [14].

Quality of life (QoL) may diminish in patients with severe cases of onychophagia, in which nail biting is frequent and causes significant psychological distress and considerable physical damage. Onychophagia is an unwanted habit and affected patients usually have made multiple attempts to stop but are unsuccessful in doing so. A questionnaire-based study of 339 medical students analyzed the influence of onychophagia on QoL and stigmatization. Nail biters had significantly higher QoL impairment scores compared to non-biters. Inability to stop biting (23.3 vs. 10; *p* < 0.01), visible nail deformities (19.2 vs. 10; *p* = 0.03), increased time spent on biting (*ρ* = 0.28; *p* = 0.02), and fingernail involvement (*ρ* = 0.26; *p* = 0.03) contributed further to QoL impairments. The level of stigmatization for nail biters was higher compared to non-biters (0.6 ± 1.2 vs. 0.2 ± 0.6 points, respectively; *p* < 0.01) [13]. In young children, social and family pressures to stop nail biting may negatively impact QoL and induce behavioral and emotional problems [8].

Onychophagia is closely linked to high stress levels. In a cross-sectional study measuring the stress levels using a Perceived Stress Scale in university students with onychophagia, the median score was significantly higher for nail biters (29) compared to non-biters (28; *p* = 0.001). Median QoL score was significantly lower for nail biters (26) compared to non-biters (28) (*p* < 0.001) [10].

#### 3.1.6. Physical Exam

Although onychophagia is a common condition, perceived stigma may cause treatment delays, as patients may feel shame and avoid medical evaluation. Patients will rarely present with nail biting as a primary complaint, making it more challenging for physicians to diagnose onychophagia. Observation for nail biting in the examination room, thorough questioning of the patient’s nail habits in a non-judgmental way, and physical examination are important to identify onychophagia in the earlier stages.

Routine inspection of all twenty nail units is recommended for diagnosing onychophagia. A full-body skin examination, including the scalp and secondary hair, is performed to look for other evidence of other BFRBs (e.g., skin picking, hair pulling, nail picking), as they may coexist. Chronic nail biters are more likely to suffer from orodental abnormalities; therefore, inspection of the oral mucosa must not be overlooked.

**Figure 2:**
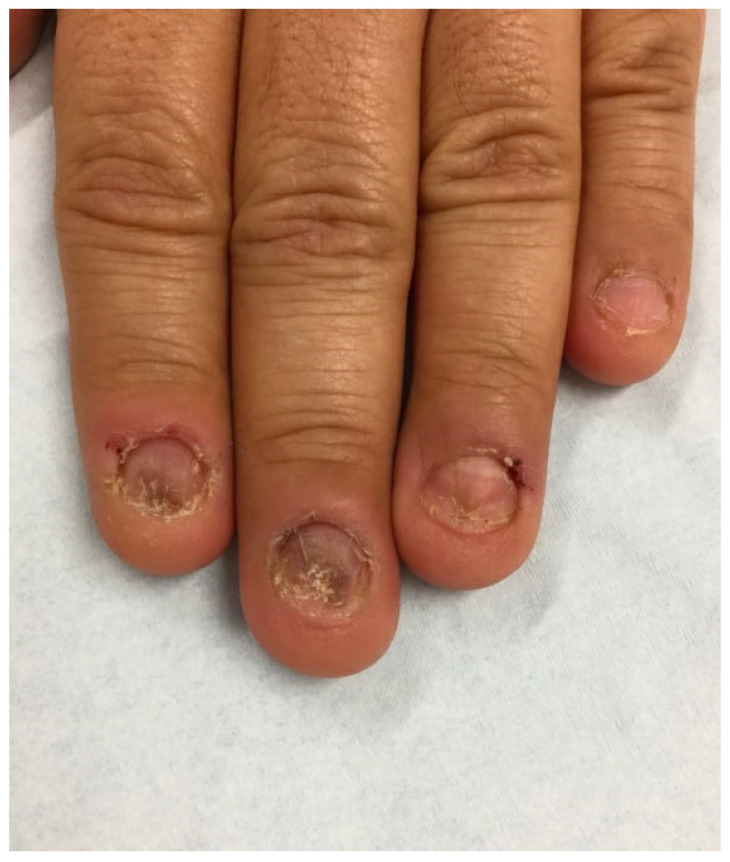
A 46-year-old nail biter with short, uneven nails and ragged cuticles. Nail folds are erythematous and in different stages of healing.

Patients typically present with abnormally short and uneven nails, absent or ragged cuticles, and nail folds in different stages of healing (Figure 2) [1,15]. Other visible changes in the nail and periungual regions include linear and pinpoint hemorrhages, longitudinal melanonychia, transverse grooves, brittleness, macrolunula, and pterygium, a scar in the nail matrix [16]. Dermatoscopic evaluation of onychophagia shows loss of nail plate with ragged distal nail borders [16]. Onychophagia usually occurs in the fingernails, as toenails are rarely bitten [3]. Because toenails are physically harder to bite compared to fingernails, toenail biting suggests possible psychiatric comorbidities [9].

Onychophagia is relatively easy to diagnose clinically. Histopathological analysis is not necessary for diagnosing onychophagia; however, it may be warranted when other diagnoses are being considered. Onychophagia may mimic other nail conditions such as nail psoriasis, nail lichen planus, onychotillomania (nail picking), and chronic paronychia [1,4]. A nail biopsy with histopathology would reveal findings consistent with trauma, such as entrapped red blood cells and focal hyperkeratosis [1].

#### 3.1.7. Complications

Nail-biting complications are not limited to the nail plate, and may affect the periungual region and oral cavity. In chronic nail biters, partial or complete loss of the nail plate can occur, exposing the nail bed. As a result, the nail bed keratinizes, leading to irreversible nail plate shortening [16].

Chronic trauma to the nail matrix may lead to melanocytic activation, presenting clinically as longitudinal melanonychia. Gray-brown longitudinal bands of variable width may involve one or more nail plates [16,17]. Nail biting can also predispose patients to secondary infections. Acute paronychia is typically due to inoculation of bacteria from the mouth to the fingers [18]. The soft tissue surrounding the nail bed becomes erythematous, warm, and tender, with risk of developing into an abscess or, rarely, osteomyelitis [19,20]. Chronic paronychia presents with loss of cuticle and nontender erythema of the nail folds, but is not due to bacteria, unlike acute paronychia. Other periungual infections include herpetic whitlow and subungual warts [21,22].

Oral and dental complications include gingival injury, leading to swelling and abscess, increased incisor wear, malocclusion of teeth, apical root resorption, and rotation of the incisors [1,13]. Recognition of these symptoms should prompt a referral to a dentist for further evaluation. Pain and dysfunction of the temporomandibular joint have also been reported in chronic nail biters [23]. Patients with onychophagia have a higher oral bacterial burden. Colonization of the oral cavity by the *Enterobacteriaceae* family, specifically *Enterobacter* spp. and *Escherichia coli*, is frequently seen in nail biters [18,24]. This predisposes nail biters to local and systemic infections if there is oral trauma or when enteric bacteria are ingested.

#### 3.1.8. Treatment

While nail biting is a difficult behavior to modify, a multidisciplinary approach can effectively manage onychophagia. Stimulus control, habit reversal training (HRT), and pharmacotherapy alone, or more commonly in combination, is frequently used for treatment (Table 1).

Stimulus control procedures involve reducing outside triggers (e.g., splintered cuticles) or cues (e.g., stress, idleness, overstimulation), making nail biting physically difficult, and removing positive reinforcements (aversion therapy) [31]. Preventative nail filing and trimming reduce the appearance of splintered cuticles to decrease nail-biting temptations. Professional manicures for men and women can reduce nail biting for patients who are motivated to preserve their manicure, and also serve to hide the dystrophic nails while the affected nails are growing out [31]. Gloves or bandages around the fingers are physical barriers that make biting more difficult. However, patients may find these unacceptable options, since they may interfere with daily activities and reduce sensations in the fingers. They may also worsen feelings of embarrassment toward nail biting when worn in social settings.

Aversion therapy refers to the repeated pairing of an unwanted behavior with discomfort to break the habit [31]. For nail biters, the application of an unpleasant-tasting polish to the nails interferes with the enjoyable aspect of biting. Aversion therapy is discouraged in younger children as it may induce opposition, leading to increased nail biting to attract attention [32,33]. The success of aversion therapy is dependent on consistent reapplication of the polish. In patients struggling to remember to reapply polish, a nonremovable reminder (NrR) can be used as an alternative to aversion therapy. In a study of 80 nail biters, where half was treated using NrRs and the other half a bitter-tasting polish, the NrR group had a lower drop-out rate (12% vs. 26%) compared to the mild aversion therapy group, but both therapies were equally effective in reducing nail biting, including all participants who started the study (Wilks’s lambda: F2.59 = 110.94; *p* < 0.0001). However, when considering only non-dropouts, mild aversion was more effective (Wilks’s lambda: F2.59 = 3.35; *p* < 0.042). In addition, the results were sustainable, since nail-biting scores were lower at five months after the last therapy session than scores at baseline (*t*(42) = 8.05; *p* < 0.0001). Overall, NrRs are effective options for patients with aversion therapy noncompliance and may promote long-term behavior changes in nail biting [25].

HRT is a technique that may be helpful in treating onychophagia. Initially described to treat tics and nervous habits, HRT involves three components: awareness training, competing response training, and social support [31,34]. Awareness training brings the habit into consciousness by having the patient write or say aloud their triggers for nail biting and its negative consequences. Competing response training occurs once nail biting is brought into awareness. Patients learn alternative behaviors like fist clenching, clapping, or sitting on hands when they have the urge to bite [35].

Several controlled trials have studied the effects of HRT in treating onychophagia [26,27,36]. In a clinical trial of 30 adult nail biters comparing the efficacy of HRT to placebo using pre- and post-treatment nail length measurements, post-treatment nail lengths were significantly longer in the HRT group (12.1 ± 1.9 mm) compared to the placebo group (8.8 ± 1.6 mm) (F = 21.2, df = 1.22; *p* < 0.01) [26]. In another study of 97 adult nail biters, participants were randomized into the HRT group (*n* = 45) or negative practice group (*n* = 52), in which subjects simulated nail biting and told themselves how ridiculous their habit appeared. Nail-biting episodes decreased by 99% in the HRT group compared to a 60% reduction in the negative habit group (*p* < 0.001) [27].

One clinical trial compared the effectiveness of mild aversion therapy to HRT using a fist-clenching competing response. Although mild aversion and competing response techniques were both effective in reducing nail biting (*p* < 0.01), the latter resulted in decreased nail fold erosions (p < 0.05) and severity of nail biting (*p* < 0.01) and increased participants’ feelings of nail-biting control (*p* < 0.01) compared to the aversion therapy group [28].

A support system (i.e., a family member or other person also trying to break their own habit) can also keep the patient accountable. These individuals regularly remind patients to stop biting and encourage the use of competing responses. Patients may also enroll in group therapy programs. The TLC Foundation for Body-Focused Repetitive Behaviors is a national organization that connects patients to other chronic nail biters in their region [37].

Pharmacotherapy is a second-line treatment for nail biting [31]. Currently, no drugs are approved by the Food and Drug Administration for treating BFRBs; however, some medications are helpful in managing onychophagia. There is a growing interest in using N-acetylcysteine (NAC) to treat BFRBs. NAC is a glutamate modulator that has been used in clinical trials on impulse control disorders, including onychophagia. In a double-blind, randomized clinical trial of 42 children and adolescents with onychophagia, nail length increased after treatment with 800 mg/day of NAC over a one-month period (5.21 mm) compared to placebo (1.18 mm; *p* < 0.04). However, there was no significant difference between both groups after two months of NAC treatment. One patient in the NAC group reported headache, agitation, and social withdrawal, while another experienced severe aggression during NAC treatment. No adverse effects were noted in the placebo group [29].

The tricyclic antidepressant (TCA), clomipramine (mean dose, 120 ± 48 mg/day), was superior to desipramine (mean dose, 135 ± 53 mg/day) in treating onychophagia in a 10-week double-blind cross-over trial of 25 patients. Based on three clinical biting scales (nail biting severity, nail biting impairment, and clinical progress), there was a greater decrease in biting in the clomipramine group compared to the desipramine group (F = 3.75, *p* < 0.04; F = 5.27, *p* < 0.02; F = 7.65, *p <* 0.01, respectively). Associated adverse effects of both treatment drugs included dry mouth, fatigue, insomnia, constipation, sweating, and dizziness, requiring 11 subjects (44%) to drop out of the study. A two-fold increase in serum alanine aminotransferase levels was also reported in one patient taking clomipramine, which resolved after treatment discontinuation [30].

Use of selective serotonin reuptake inhibitors (SSRIs), bupropion, and lithium to treat onychophagia was successful in single case reports [38,39,40,41]. Two nail biters with coexisting depression and bipolar disorder, treated with bupropion and lithium, respectively, had improvements in both their onychophagia and their respective psychiatric disorders. However, SSRIs should be prescribed with caution, as exacerbation of impulse-related disorders have been reported [32,42].

Despite these promising results, there is limited evidence on the efficacy of using NAC and antidepressants to treat onychophagia. Larger clinical trials are necessary to determine the effective dosage and treatment protocol for their use.

### 3.2. Onychotillomania

#### 3.2.1. Overview

Onychotillomania is defined as repetitive picking or pulling of the nail unit, causing damage to the nail matrix, nail bed, nail plate and periungual skin. This behavior is self-induced using the patient’s own fingers and nails, though tools (e.g., scissors, nail files, knives) can also be used for nail manipulation [43]. Onychotillomania is grouped with other BFRBs, including onychophagia, trichotillomania, and excoriation disorder. A variant of onychotillomania is habit tic, a nervous habit characterized by repetitive rubbing, picking, and pushing back the cuticle [44]. Other related nail behavioral disorders include onychotemnomania (cutting nails extremely short), onychoteiromania (excessive nail rubbing until they become very thin), and onychodaknomnaia (nail biting causing painful pleasure) [45,46].

#### 3.2.2. Prevalence

The true prevalence of onychotillomania is unknown and likely underreported. Mild nail picking is relatively common in the general population, but only a small percentage experience considerable distress from excessive nail picking [15,43]. In a cross-sectional study assessing for onychophagia and onychotillomania in 339 Polish medical students, 160 cases of onychophagia were noted, while only three cases of onychotillomania were reported, corresponding to a prevalence of 0.9%. The three students who reported nail picking (two females and one male) had a mean age of onset of 8.6 ± 2.3 years and duration of 14 ± 2.5 years [2].

#### 3.2.3. Psychiatric Classification

Onychotillomania has not been fully classified in the DSM-5. Like onychophagia, the DSM-5 does not include onychotillomania as a separate diagnosis. Nail picking may be categorized under “Other Specified Obsessive-Compulsive and Related Disorders”, specifically the BFRB disorders subsection [7]. Onychotillomania has received less attention in the psychiatric literature compared to similar self-induced dermatological disorders, including trichotillomania and excoriation disorder [4]. Nail picking is frequently omitted from BFRB questionnaires and participants with onychotillomania have been excluded from studies measuring validation of skin picking on a reward scale [43,47]. Onychotillomania is underrecognized and underreported, likely because it is not uniformly recognized as a diagnosis in the medical literature.

#### 3.2.4. Associated Disorders

Psychiatric disorders are often comorbid with onychotillomania, namely depression, anxiety, and psychosis. In 29 onychotillomania cases, eight were associated with depression with or without psychosis [48,49,50,51,52,53,54], four with general anxiety disorder, adjustment disorder, or specific phobias [2,55,56], and two with psychosis and hypochondrial delusions [57,58]. In one case, there were no psychiatric comorbidities, and for the remaining 14 cases no psychiatric evaluations were performed [59,60,61,62,63].

Nail picking is also associated with high levels of dissociation where patients have fragmented or no memory of their self-inflicted trauma. Higher levels of dissociation have been linked to more severe BFRBs, and onychotillomania has been associated with completed suicide in one case report [50].

Certain rare congenital disorders have also been associated with onychotillomania. Smith–Magenis syndrome is a neurodevelopmental disorder characterized by developmental delay, intellectual deficiency, dysmorphic facial features, and behavioral abnormalities, including nail picking, finger and hand biting, head banging, and aggression [45,64]. Onychotillomania can also be seen in patients with Lesch–Nyhan syndrome, an X-linked recessive disorder caused by a deficiency in the HPRT enzyme, which is responsible for purine recycling. Absence of the enzyme results in uric acid accumulation, leading to intellectual disability and self-injurious behavior [45].

Dermatitis artefacta (DA) is a factitious disorder defined by self-inflicted injury with the intention of assuming a sick role without any external rewards [4]. Individuals with Munchausen syndrome may have coexisting DA; however, nail and skin involvement are rarely seen in Munchausen syndrome [65]. Patients with DA present with self-induced lesions that can mimic a variety of dermatologic disorders, including onychotillomania. The lesions are typically geometric and surrounded by normal-appearing skin [4]. DA should be considered when patients present multiple times for the same problem, clinical findings are atypical in distribution and morphology, or the lesions do not respond to standard treatment [4,66].

#### 3.2.5. Clinical History

The diagnosis of onychotillomania is made clinically. Patients may report feelings of tension before and relief after nail picking. For some, nail picking is an unconscious behavior, leading many patients to deny their habits from poor insight [2,43]. Patients who are fully aware of their nail picking behavior usually report multiple prior attempts to stop and are apologetic about their unwanted habits [67]. Nail picking is rarely seen as the primary complaint and patients are often diagnosed incidentally during routine inspection of the nails [45]. Patients with coexisting psychiatric conditions (i.e., obsessive compulsions, delusions of infestation) may be diagnosed a psychiatrist and referred to a dermatologist for their nail changes. Understanding the patient’s awareness of their nail-picking behavior, as well as psychiatric history, can direct the approach to treatment.

#### 3.2.6. Physical Exam

**Figure 3:**
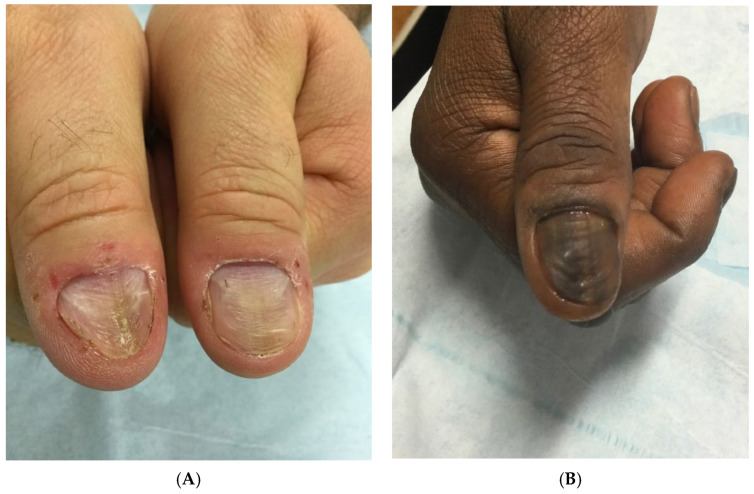
(**A**) A 58-year-old nail picker with habit tic deformity of the bilateral thumbnails. There are parallel transverse grooves in the nail plate. (**B**) A 54-year-old nail picker with transverse grooves in the nail plate (habit tic deformity) and longitudinal melanonychia from chronic nail picking.

A full examination of all twenty nails, skin, scalp, and hair is warranted in patients with a suspected diagnosis of onychotillomania and to assess for other coexisting BFRBs. For patients who are not aware or deny nail picking, careful observation for nail picking during the visit can provide evidence for the diagnosis [45]. An abbreviated psychiatric examination is also recommended, as onychotillomania is associated with coexisting depression, anxiety, and psychosis. The clinical manifestations of onychotillomania are primarily depicted in case reports with limited definitions for symptoms and signs of disease [68]. Clinical findings are often asymmetric, nonspecific, and bizarre-appearing. Common nail plate abnormalities include transverse ridges, thinning, macrolunula, and generalized dystrophy. Repetitive picking of the proximal nail folds can traumatize the nail matrix, resulting in nail plate ridging. A characteristic nail finding associated with onychotillomania is the habit tic deformity, characterized by multiple parallel transverse grooves, most commonly affecting the thumbnails (Figure 3) [4]. The periungual skin, particularly the nail folds and cuticles, can also be affected and can appear erythematous and tender, with crusts and erosions [43].

Dermatoscopic evaluation is helpful in distinguishing onychotillomania from other nail conditions. In a study characterizing the most common dermatoscopic features of onychotillomania in 36 patients, 69.4% had wavy lines (uneven longitudinal lines in different planes with a wavy appearance from uneven nail plate growth), 63.9% obliquely oriented nail bed hemorrhages, and 47.2% nail bed gray discoloration. These changes were not seen in nail lichen planus, nail psoriasis, or onychomycosis [68].

A nail biopsy with histopathological analysis is rarely required for diagnosis for onychotillomania. However, it may be necessary in cases of severe onychodystrophy, without historical clues. Differential diagnoses include onychophagia, nail lichen planus, nail psoriasis, onychomycosis, acute and chronic paronychia, 20-nail dystrophy, and epidermolysis bullosa aquita [4,45]. Pathological findings are often nonspecific, but can rule out other nail conditions. There is typically epithelial hyperplasia, acanthosis, and hyperkeratosis [43,45]. Lichen simplex chronicus, or a thickening of the skin from repetitive rubbing or scratching, may also be seen [59].

#### 3.2.7. Complications

Long-term picking and manipulation of the nail unit can result in permanent nail dystrophy. Trauma to the proximal nail folds can cause melanocytic activation of nail matrix melanocytes, resulting in longitudinal melanonychia. These changes are usually permanent and do not reverse with the cessation of nail-picking behavior [63]. Onychotillomania can be complicated by bacterial and viral infections, including acute bacterial paronychia, herpes simplex virus, and human papilloma virus [45]. Chronic paronychia may also result from frequent manipulation of the nail unit. In severe cases of nail picking, pterygium, or anonychia, or complete loss of nail, can occur [43,48].

#### 3.2.8. Non-Pharmacological Treatment

Onychotillomania can be managed non-pharmacologically or pharmacologically. Supportive measures for treating onychotillomania are typically recommended as first-line therapy. Use of band aids and occlusive dressings have been efficacious in some cases [61,62]. However, patients may find these bandages socially unacceptable or burdensome and therefore noncompliant. Another option is cyanoacrylate adhesive, also known as ‘super glue’. The product is applied once or twice a week over the cuticles of the affected nails, which serves as a protective barrier and a reminder to stop picking. In a case report of two patients using cyanoacrylate adhesives to treat onychotillomania, both patients ceased nail picking within three to six months and achieved normal-appearing nails [69].

Behavioral modification using cognitive behavioral therapy (CBT) may increase awareness of nail picking [70]. Since CBT has been used successfully to treat BFRBs, it may also be helpful for onychotillomania treatment. For example, in a clinical trial (*n* = 16) using a CBT protocol originally designed for trichotillomania to treat skin picking disorder, 63% and 52% patients improved with individual and group CBT treatment, respectively [71]. Another behavioral therapy used to treat onychotillomania is HRT. In one case report of a patient with onychotillomania treated with HRT, there was a reduction in nail-picking frequency from 8–10 h/day to 0–5 min/day, increased nail length, improved finger sensitivity, and less shame and embarrassment after 21 weekly sessions. [49].

Given the potential comorbid psychiatric illnesses with onychotillomania, referral to a psychiatrist may be necessary. Dermatologists should inquire about intrusive thoughts, compulsions, and impact on QoL to determine if further psychiatric evaluation is warranted [43]. A multidisciplinary approach to treatment can benefit patients who experience significant psychological distress.

#### 3.2.9. Pharmacological Treatment

Results of pharmacologic treatment for onychotillomania are limited to a few case reports, as no large randomized controlled studies have been performed. NAC, which has not been studied for onychotillomania, is helpful for other BFRBs, so may be considered for onychotillomania management. Psychotropic drugs, including SSRIs, TCAs, and typical antipsychotics have been successful in treating nail picking; however, patients had comorbid psychiatric illness such as depression, OCD, or psychosis.

SSRIs and TCAs are not first-line treatments for nail picking, but may be beneficial in cases of comorbid depression or anxiety. In a case of onychotillomania with coexisting depression, one patient was treated with sertraline 150 mg/day. After four weeks, the patient’s depressive symptoms improved, with decreased nail picking, and resolution of both conditions at six weeks [51]. Amitriptyline 50 mg/day improved nail picking in a patient with depressive neurosis after an unknown period of time [53].

Antipsychotics can be used to treat nail picking in patients with comorbid psychosis. One patient with nail picking and comorbid trichotillomania and delusions of infestation was treated successfully with 200 mg/day thioridazine. After one month of treatment, the patient had significant improvement in both psychiatric conditions and onychotillomania [53]. In a patient with onychotillomania and fixed hypochondrial delusions that she had an unknown nail disease, pimozide (Orap) successfully treated the delusions, resulting in normal-appearing nails after seven months [57].

In one case report, a 19-year-old woman with onychotillomania was treated with monthly intramatrical nail injections of triamcinolone acetonide (0.2 mL of 5 mg/mL) for 3 months, followed by bimonthly injections for 6 months and daily topical calcipotriol/betamethasone dipropionate. Nail appearance improved after three months, with completely normal nail folds, cuticles, and nail plates by eight months [55].

## 4. Conclusions

Onychophagia and onychotillomania are BFRBs that are often underrecognized in the clinical setting. Patients rarely present with nail biting or picking as the primary complaint, making these nail disorders challenging to diagnose. These nail conditions may be comorbid with other psychiatric conditions and require a multidisciplinary approach to properly diagnose and manage. Nail biters and nail pickers may experience considerable psychosocial consequences from their habits. Currently there are no standardized treatments for onychophagia and onychotillomania. Larger clinical trials are necessary to develop effective therapies for both conditions.

## Figures and Tables

**Table 1:** Summary of onychophagia treatment studies.

Behavior Modification Treatment Studies						
Study	Design	Materials and Measures	Number of Subjects (M, F)	Subject Demographics, Mean Age, (Age Range)	Results	Conclusions
Koritzky and Yechiam (2011) [25]	Randomized comparative study on the effectiveness of using nonremovable wristbands (*n* = 40) vs. mild aversion therapy (applying bitter-tasting polish twice a day) (*n* = 40) for six weeks. Removal of wristband or discontinuation of polish disqualified participant.	Malone–Massler scale for nail biting severity at start of study, then three weeks, six weeks, and five-month follow-up.	80 (51, 29)	Adults, 25, (19–41)	The NrR group had a lower drop-out rate (12% vs. 26%; *p* < 0.06) and was equally effective as aversion therapy when considering all participants (Wilks’s lambda: F2.59 = 110.94; *p* < 0.0001). Aversion therapy was more effective when considering only non-dropouts (Wilks’s lambda: F2.59 = 3.35; *p* < 0.042). Lower nail-biting scores were maintained five months post study completion compared to scores at the start of the study (*t*(42) = 8.05; *p* < 0.0001).	NrRs can be used as an alternative treatment for patients who have noncompliance with aversion therapy. NrRs may produce lasting change in nail-biting behavior.
Twohig et al. (2003) [26]	Randomized clinical trial on the effectiveness of HRT (awareness training, competing response training, and social support) (*n* = 15) vs. placebo control (nail biting discussions) (*n* = 15) for two hours over three sessions.	Nail length measurements (mm) taken before treatment, after treatment, and at five-month follow-up.	30 (7, 23)	Adults, 21.5, (18–49)	With HRT there was a 22% increase in nail length, compared to 3% for placebo. Differences in nail length were significantly different in the HRT group (12.1 ± 1.9 mm) with longer nail lengths than the placebo group (8.8 ± 1.6 mm; F = 21.2, df = 1.22; *p* < 0.01). At the five-month follow-up, the HRT group (11.72 ± 2.5 mm) maintained a 19% increase in nail length from pretreatment measurements compared to 0% in the placebo group (8.5 ± 1.7 mm; F = 7.8, df = 1.17; *p* < 0.05).	HRT is an effective intervention for treating onychophagia with long lasting changes.
Azrin, Nunn, and Frantz (1980) [27]	Randomized clinical trial on the effectiveness of HRT (awareness training, competing response training, and social support) (*n* = 45) vs. negative practice (subjects simulate nail biting and tell themselves how ridiculous the habit appears) (*n* = 45) for five months after one two-hour training session.	Number of nail biting episodes self-recorded by subjects every day for five months.	97 (38, 59)	Adults, HRT: 28 (11–56), negative practice: 31 (11–64)	Number of nail biting episodes decreased by 99% (10 to 0.3× per day) in the HRT group compared to a 60% (12 to 4× per day) reduction in the negative practice group (*p* < 0.001). 40% of HRT and 15% of negative practice participants completely stopped nail biting by the end of the study.	HRT was more effective than the negative practice treatment in reducing frequency of nail biting.
Silber and Haynes (1992) [28]	Clinical trial comparing mild aversion therapy (applying bitter-tasting polish twice a day) (*n* = 7) vs. use of competing response (fist clenching) (*n* = 7) vs. control (nail-biting monitoring and positive encouragement) (*n* = 7) for three weeks after one week of baseline self-monitoring to increase awareness of nail-biting habit.	Nail length measurements (mm), nail fold erosion scale, Malone–Massler scale for nail biting severity, and self-control questionnaire at start of study and at four weeks.	21	Adults, mild aversion: 21, competing: 24, control: 22	Both aversion therapy and competing response showed improvements in nail length (F1.18 = 26.27; *p* < 0.01). The competing group had decreased nail fold erosions (U7.7 = 8.50; *p* < 0.05), decreased severity of nail biting (U7.7 = 4.00; *p* < 0.01), and increased feeling of control (U7.7 = 4.50; *p* < 0.01) compared to aversion therapy group.	Aversion therapy and competing response techniques are effective in treating onychophagia. The competing response showed more beneficial effects in treating nail biting compared to aversion therapy.
**Pharmacological Treatment Studies**						
Ghanizadeh et al. (2013) [29]	Double-blind, randomized, placebo-controlled clinical trial investigating use of 800 mg/day NAC (*n* = 21) vs. placebo (*n* = 21) for two months.	Nail length measurements (mm) taken before treatment, one month after enrollment, and two months after enrollment.	42 (14, 28)	Children and adolescents, NAC: 9.28, placebo: 10.76, (6–18)	Patients taking 800 mg/day NAC had significantly increased nail length (5.21 mm) after one month compared to placebo (1.18 mm; *p* < 0.04). No significant difference was observed after two months. Adverse effects included headache, agitation, social withdrawal, and severe aggression.	NAC decreases nail biting behavior in children and adolescents over the short term.
Leonard et al. (1991) [30]	Double-blind, cross-over trial comparing clomipramine hydrochloride (mean dose, 120 ± 48 mg/day) vs. desipramine hydrochloride (mean dose, 135 ± 53 mg/day) for 10 weeks (five weeks clomipramine + five weeks desipramine) after two-week single-blind placebo.	Nail Biting Severity Scale, Nail Biting Impairment Scale, and Clinical Progress Scale at baseline and weekly until 12 weeks.	25 (6, 19)	Adults, 32.7, (21–42)	There was a greater decrease in nail biting with clomipramine treatment than with desipramine as measured on the Nail Biting Severity (F = 3.75, df = 1.12; *p* < 0.04), the Nail Biting Impairment (F = 5.27, df = 1.12; *p* < 0.02), and the Clinical Progress (F = 7.65, df = 1.12; *p* < 0.01) scales. Adverse effects included dry mouth, fatigue, insomnia, constipation, sweating, dizziness, and abnormal liver enzymes.	Clomipramine decreases nail biting more than desipramine as measured on three clinical biting scales.

Abbreviations: NrR, nonremovable reminder; HRT, habit reversal training; NAC, N-acetylcysteine.

## Data Availability

Not applicable.
